# Contribution of central sensitization to stress-induced spreading hyperalgesia in rats with orofacial inflammation

**DOI:** 10.1186/s13041-020-00645-x

**Published:** 2020-07-28

**Authors:** Jia-Heng Li, Jia-Le Yang, Si-Qi Wei, Zhuo-Lin Li, Anna A. Collins, Min Zou, Feng Wei, Dong-Yuan Cao

**Affiliations:** 1 Key Laboratory of Shaanxi Province for Craniofacial Precision Medicine Research, Research Center of Stomatology, Xi’an Jiaotong University College of Stomatology, 98 West 5th Road, Xi’an, Shaanxi 710004 People’s Republic of China; 2 Department of Orthodontics, Xi’an Jiaotong University College of Stomatology, 98 West 5th Road, Xi’an, Shaanxi 710004 People’s Republic of China; 3 Department of Neural and Pain Sciences, University of Maryland School of Dentistry; the UM Center to Advance Chronic Pain Research, 650 West Baltimore Street, Baltimore, MD 21201 USA

**Keywords:** Hyperalgesia, Stress, Comorbidity, Central sensitization, Orofacial inflammation

## Abstract

Temporomandibular disorder (TMD) is commonly comorbid with fibromyalgia syndrome (FMS). The incidence of these pain conditions is prevalent in women and prone to mental stress. Chronic pain symptoms in patients with FMS and myofascial TMD (mTMD) are severe and debilitating. In the present study, we developed a new animal model to mimic the comorbidity of TMD and FMS. In ovariectomized female rats, repeated forced swim (FS) stress induced mechanical allodynia and thermal hyperalgesia in the hindpaws of the 17β-estradiol (E2) treated rats with orofacial inflammation. Subcutaneous injection of E2, injection of complete Freund’s adjuvant (CFA) into masseter muscles or FS alone did not induce somatic hyperalgesia. We also found that the somatic hyperalgesia was accompanied by upregulation of GluN1 receptor and serotonin (5-hydroxytryptamine, 5-HT)_3A_ receptor expression in the dorsal horn of spinal cord at L4-L5 segments. Intrathecal injection of N-methyl-D-aspartic acid receptor (NMDAR) antagonist 2-amino-5-phosphonovaleric acid (APV) or 5-HT_3_ receptor antagonist Y-25130 blocked stress-induced wide-spreading hyperalgesia. These results suggest that NMDAR-dependent central sensitization in the spinal dorsal horn and 5-HT-dependent descending facilitation contribute to the development of wide-spreading hyperalgesia in this comorbid pain model.

## Introduction

Functional pain syndromes such as temporomandibular disorder (TMD), fibromyalgia syndrome (FMS), irritable bowel syndrome (IBS), and chronic pelvic pain are an enormous global health problem. Clinical observation indicates that these debilitating pain conditions often occur concomitantly or overlap finally. They characterize common features such as significant prevalence rate in women and relationship with varying affective or cognitive influences [1, 2]. Our previous studies showed that the mild stress with 3 day repeated forced swim (FS) induced prolonged visceral hypersensitivity in female rats with orofacial muscle inflammation, providing a preclinical animal model to investigate the mechanisms of the comorbid visceral pain like clinical IBS in patients with TMD [1, 2]. Regarding the fact that FMS patients are likely to suffer from other concurrent pain conditions such as TMD or headaches in clinic [3], the primary purpose of the present study was to clarify whether widespread somatic hyperalgesia also develops in animals with orofacial pain after stress, a typical characteristic observed in patients with comorbid TMD and FMS.

Different from tissue or nerve injury-induced pain, comorbid pain conditions displayed in the organ system are the lack of structural or disease etiology. Although their biological mechanism is still less understood, comorbid pain conditions including FMS have been reported to heavily rely on a myriad of environmental influences and genetic susceptibility [4]. Interestingly, functional magnetic resonance imaging (fMRI) has demonstrated an increased response to stimuli in central pain pathways of the patients with FMS [5]. Accumulating pre-clinical evidence suggests that involvement of upregulated N-methyl-D-aspartic acid (NMDA) receptors in the spinal dorsal horn and spinal serotonin (5-hydroxytryptamine, 5-HT)_3_ receptor-mediated descending pain facilitation maintains hyperalgesia and allodynia after tissue and nerve injury [6–8]. These studies provide benefit by narrowing the focus on central sensitization in the spinal cord as a possible mechanism underlying the development of comorbid pain conditions and will facilitate effective treatment in future clinical studies. Thus, we further conducted Western blot analysis to examine the expression of NMDA receptors and 5-HT_3_ receptors in the lumbar spinal cord of rats with orofacial inflammation followed by stress, and utilized pharmacological approaches to validate their contribution to the development of stress-induced widespread pain.

There is increasing evidence for sex or gender difference in pain sensitivity and intervention [9]. The prevalence of functional or comorbid pain conditions are great for women than men [10]. Notably, steroid hormones including estradiol are key players in sex difference of pain. Therefore, ovariectomy and estrogen replacement in rodent model were used in order to ablate the rapidly cycling hormone production and mimic the fluctuation of normal estrous cycle. On this basis, we examined the effects of ovariectomy on stress-induced widespread pain in female rats with orofacial inflammation.

## Material and methods

### Animals

Adult female Sprague-Dawley rats (225–250 g) were obtained from Xi’an Jiaotong University Laboratory Animal Center (Xi’an, Shaanxi, China) and housed with a 12-h light-dark cycle. Food and water can be obtained freely. Experimental protocols were approved by the Institutional Animal Care and Use Committees. In addition, we abided by the guidelines of the International Association for the Study of Pain.

### Experimental design

**Fig. 1:**
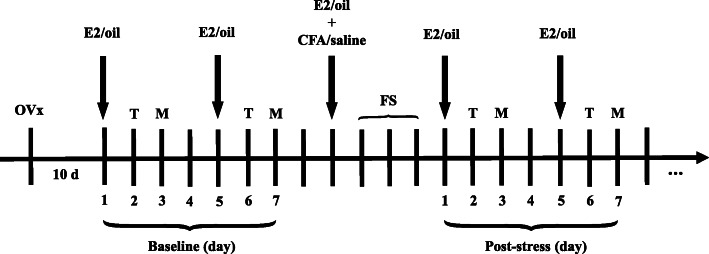
Experimental design. All female rats were OVx. After 10 days of recovery, E2 or oil was injected subcutaneously every 4 day. The baselines of thermal withdrawal latency (T) and mechanical withdrawal threshold (M) were tested before FS. One day before FS, bilateral masseter muscles were injected with CFA. Three day repeated FS was applied after injection of CFA. From day 2 post last FS, thermal withdrawal latency and mechanical withdrawal threshold were measured on the second and third days, respectively, after each E2/oil injection. The measurements were continued until the thermal withdrawal latency or mechanical withdrawal threshold returning to the baseline level. OVx, ovariectomized; E2, estradiol; FS, forced swim; CFA, complete Freund’s adjuvant

Under anesthetized with isoflurane inhalation (3%), rats were ovariectomized (OVx) with a dorsolateral approach as previous descripted [2]. The experimental protocol was shown in Fig. 1. OVx rats were injected subcutaneously with 17β-estradiol (E2; 50 μg in 100 μl safflower oil) at 4 day intervals to mimic the fluctuation of a normal estrous cycle of rats so that E2 levels in these rats were similar at the same time. Orofacial inflammation was induced by injection of complete Freund’s adjuvant (CFA, 150 μL, 1:1 in saline) into the bilateral masseter muscles, which induced orofacial hyperalgesia for a week [2]. Subcutaneous injection of safflower oil and injection of saline into masseter muscles as the control of E2 and CFA, respectively. Subchronic stress was induced by a 3 day FS paradigm starting the following day of CFA injection. Non-FS rats were remained in their cages as sham treatment. In order to identify possible widespread pain, thermal withdrawal latency on the right hindpaw and mechanical withdrawal threshold on the left hindpaw were measured 1 day and 2 day after every E2/oil injection, before FS (as baseline) and after FS until thermal withdrawal latency and mechanical withdrawal threshold returning to the baseline levels. The mechanical withdrawal thresholds of the upper back and the thigh root were measured 1 day and 2 day after every E2/oil injection before FS and after FS in a separated group.

### Thermal withdrawal latency

The thermal withdrawal latency was determined using plantar thermal test device (Ugo Basile, Gemonio, Italy). Rats were placed in plastic chambers on an elevated glass floor for 30 min before testing to adapt to the environment. A movable radiant heat generator was placed below the right hindpaw beneath the glass floor. The hindpaw was stimulated by the radiant heat and the paw withdrawal latency was recorded. Cut off time was 20 s to prevent the potential hindpaw injury. The tests were repeated 3 times with 5 min intervals and the average of 3 tests was recorded as the thermal withdrawal latency on that day. The thermal withdrawal latency was measured 3 and 7 days before CFA injection and the average of two tests was considered as baseline, and following thermal withdrawal latency was tested at second day after every E2/oil injection until it recovered to baseline.

### Mechanical withdrawal threshold

According to the up and down method [11], von Frey filaments were used to measure the mechanical nociceptive threshold in three areas of the body: the left hindpaw, upper back and thigh root. Rats were placed in individual plastic chambers on an elevated platform with a surface of wire mesh for 30 min to acclimatize for the test. von Frey filaments were increased at logarithmic intervals of 0.41 to 26 g (4–255 mN). The maximum threshold was 26 g to prevent the rat’s injury [12]. Each filament was placed perpendicularly to the plantar part of the left hindpaw, or the middle upper back at T12 vertebra level and left thigh root. Stimulation was continued for 2 s. In each test, if there was no response to the filament, a stronger stimulus was then selected; if there was a positive response, a weaker one was used. Positive stimulus of the hindpaw and thigh root was recorded by flinching, lifting or licking of the hindpaw or thigh. Flinching to stimulation of the upper back was considered a positive response. Each rat was recorded 6 times and the mechanical withdrawal threshold was calculated using the threshold calculation software (JFlashDixon Calcultor, University of Arizona, USA).

### Western blot analysis

On the second day after the last FS in a separated group, rats for tissue collection were anesthetized with isoflurane (5%) and decapitated. The spinal cord rushed out with cold saline. Lumbar spinal segment (L4-L5) was taken and the dorsal part of spinal cord was isolated and stored at − 80 °C until use. Tissues were homogenized in RIPA buffer. The homogenates were centrifuged at 12,000 g for 20 min at 4 °C. The protein concentration of homogenate supernatants was measured using the bicinchoninic acid (BCA) method. Protein samples were loaded and separated by 4–12% SDS-PAGE gel and transferred onto PVDF membrane, which were blocked in blocking buffer for 2 h and then incubated with primary antibodies for 5-HT_3A_ receptor (1:300, Novus Biologicals, NB10056351, Littleton, CO, USA), GluN1 receptor (1:1000, Abcam, Ab109182, Cambridge, MA, USA) and GAPDH (1:5000, Boster, BA1054, Wuhan, China) at 4 °C overnight. After washing, the membranes were incubated with HRP-conjugated secondary anti-bodies (1:5000) for 2 h at room temperature. Bands were developed using enhanced chemiluminescence and band intensity was quantified and analyzed using ImageJ 18.0 software.

### Intrathecal injection

Rats were anesthetized with isoflurane inhalation (3%) and placed in a prone position with a round tube underneath the abdomen so that the lumbar spine was curved at the level of the L4-L5 vertebrae. A 30-gauge needle attached to a 25 μl Hamilton syringe was inserted into the tissue between the L4 and L5 spinous process at an angle of about 20°. The needle was then advanced to the groove between the spinous and the transverse processes and then moved forward the intervertebral space at an angle of about 10°. Tail flick was a sign to identify successful intrathecal injection. The NMDA receptor antagonist 2-amino-5-phosphonovaleric acid (APV, 30 nmol in 10 μl, Sigma, St. Louis, MO, USA) or 5-HT_3_ receptor antagonist Y-25130 (30 fmol in 10 ul, Tocris, Bioscience, Bristol, UK**)** was then administered at 30 min before CFA injection and each FS. The doses of drugs were determined by previous studies [13, 14] and our preliminary experiments. Intrathecal injection of saline was used for vehicle control.

### Data analysis

All data are presented as mean ± SEM. Statistical analyses were performed using GraphPad Prism 6 software. One-way ANOVA followed by Dunnett post hoc test was used for comparisons of means of the protein expression of GluN1 and 5-HT_3A_ receptors. Two-way ANOVA followed by Sidak post hoc test was used for thermal withdrawal latency and mechanical withdrawal threshold. *p* < 0.05 was considered significant.

## Results

### Repeated FS induces thermal hyperalgesia of female rats with or without orofacial inflammation

**Fig. 2:**
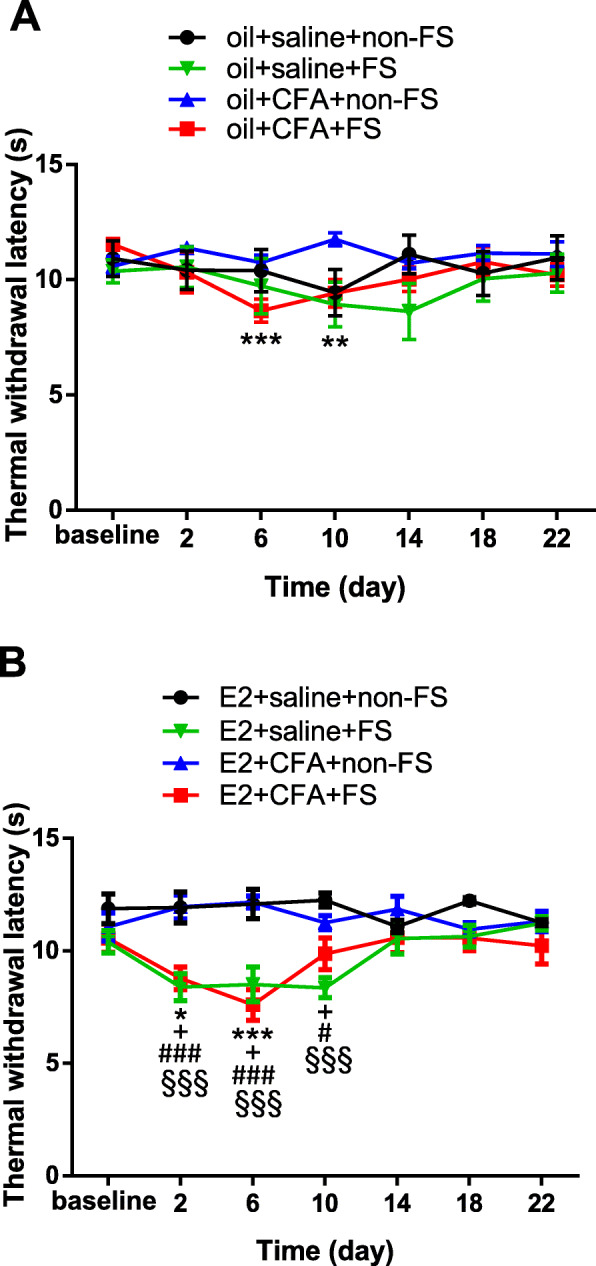
Repeated FS induced thermal hyperalgesia in the hindpaw of female rats with orofacial inflammation. **a** In oil treated groups, only rats with CFA + FS (*n* = 10) showed a decreased thermal withdrawal latency on day 6 and day 10 post FS when compared to baseline. **, *** *p* < 0.01, 0.001 vs baseline, respectively. There were not nociceptive changes in rats with FS alone (oil + saline + FS group, *n* = 9), CFA alone (oil + CFA + non-FS group, *n* = 8) or all sham treatment (oil + saline + non-FS group, *n* = 8). **b** In E2 treated groups, the thermal withdrawal latency significantly decreased on day 2 and day 6 post FS stress in the E2 + CFA + FS group (*n* = 11). *, *** *p* < 0.05, 0.001 vs baseline, respectively. FS stress produced thermal hyperalgesia for 10 days after FS (E2 + saline + FS group, *n* = 8). + *p* < 0.05 vs baseline. The thermal withdrawal latency significantly decreased on day 2 to day 10 post FS stress in the E2 + CFA + FS rats compared to the sham treated rats (E2 + saline + non-FS group, *n* = 8). #, ### *p* < 0.05, 0.001 vs the E2 + saline + non-FS group at the same time point. In rats with FS alone (E2 + saline + FS group), thermal withdrawal latency significantly decreased on day 2 to day 10 post FS stress compared to the sham group. §§§ *p* < 0.001 vs the E2 + saline + non-FS group at the same time point. No thermal hypersensitivity was observed in rats with CFA alone (E2 + CFA + non-FS group, n = 8) or sham treatment (E2 + saline + non-FS group)

To examine whether stress induces widespread pain in rats with orofacial inflammation, we first observed the individual or combined effects of E2 replacement, bilateral masseter muscle inflammation, and/or stress on thermal nociceptive threshold in the hindpaw of OVx rats. In oil treated groups, there were significant differences among groups in the thermal withdrawal latency (two-way ANOVA, *F*_18,180_ = 1.861, *p* = 0.0216 for interaction; *F*_6,180_ = 3.182, *p* = 0.0054 for time factor; *F*_3,30_ = 0.7573, *p* = 0.5269 for group factor, Fig. 2a). Sidak post-hoc tests showed that FS induced a mild thermal hyperalgesia in the hindpaw in rats with orofacial inflammation (oil + CFA + FS group, *p* < 0.001 for day 6 and *p* = 0.0038 for day 10 respectively compared to baseline, *n* = 10). There were not significant changes (*p* > 0.05 for all time points) in the thermal withdrawal latency in rats with FS alone (oil + saline + FS group, *n* = 9), CFA alone (oil + CFA + non-FS group, *n* = 8) or all sham treatment (oil + saline + non-FS group, n = 8) compared to baseline.

Similarly, significant differences in the thermal withdrawal latency among groups in E2 treated rats were also found (two-way ANOVA, *F*_18,180_ = 3.644, *p* < 0.001 for interaction; *F*_6,180_ = 3.229, *p* = 0.0049 for time factor; *F*_3,30_ = 8.818, *p* < 0.001 for group factor, Fig. 2b). Sidak post-hoc tests showed that FS stress combined with orofacial inflammation produced stronger hyperalgesia on day 2 and day 6 after FS (E2 + CFA + FS group, *p* = 0.0176 for day 2 and *p* < 0.001 for day 6 respectively compared to baseline, *n* = 11). FS alone group produced thermal hyperalgesia starting from day 2 to day 10 (E2 + saline + FS group, *p* = 0.0238 for day 2, *p* = 0.0406 for day 6, *p* = 0.0214 for day 10 respectively compared to baseline, *n* = 8). No thermal hypersensitivity was observed compared to baseline (*p* > 0.05 for all time points) in rats with CFA alone (E2 + CFA + non-FS group, n = 8) or sham treatment (E2 + saline + non-FS group, n = 8). When compared to the sham treated rats, the thermal withdrawal latency in both the FS alone group and FS stress combined with orofacial inflammation group significantly decreased (Fig. 2b). These data suggest that stress induces more robust of wide-spreading thermal hyperalgesia in the E2 treated rats with orofacial inflammation.

### Repeated FS induces wide-spreading mechanical allodynia in rats with orofacial inflammation

**Fig. 3:**
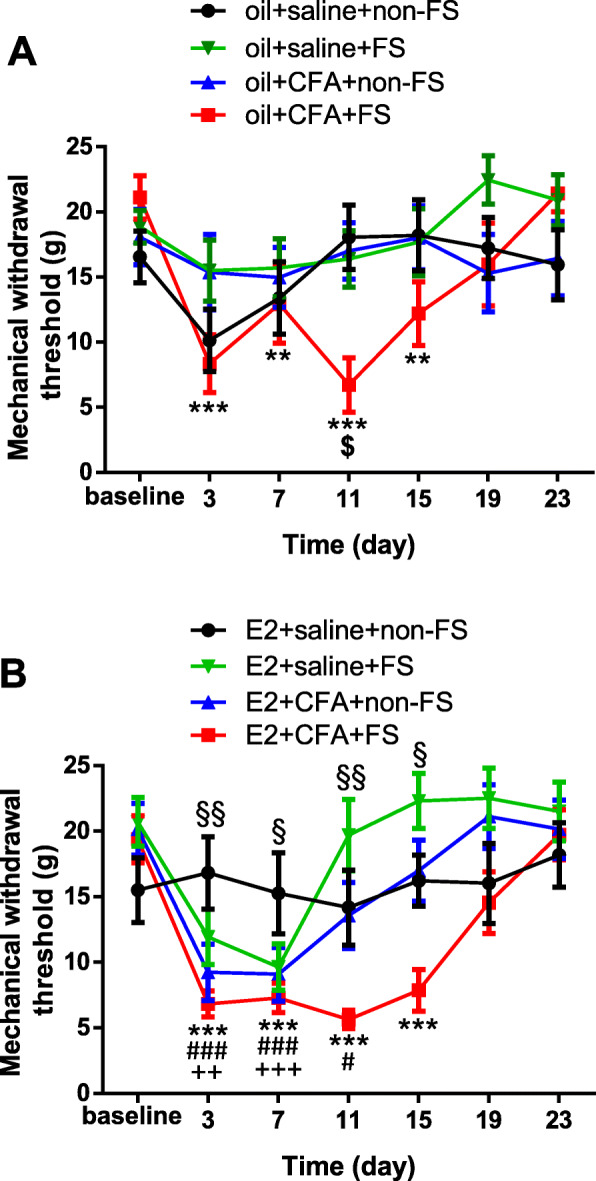
Repeated FS induced mechanical allodynia in the hindpaw of rats with orofacial inflammation. **a** In oil treated groups, only rats with CFA + FS (n = 10) exhibited mechanical allodynia lasting over 15 days post FS. **, *** *p* < 0.01, 0.001 vs baseline, respectively. The mechanical withdrawal threshold significantly decreased on day 11 post FS stress in the oil+CFA + FS rats compared with the sham treated rats. $ *p* < 0.05 vs the oil+saline+non-FS group at the same time point. There were not nociceptive changes in rats with FS alone (oil + saline + FS group, *n* = 9), CFA alone (oil + CFA + non-FS group, *n* = 8) or all sham treatment (oil + saline + non-FS group, *n* = 8). **b** In E2 treated groups, the mechanical withdrawal threshold significantly decreased for 15 days post FS stress in the E2 + CFA + FS group (*n* = 11). *** *p* < 0.001 vs baseline. FS alone produced mechanical allodynia on day 3 and day 7 after FS (E2 + saline + FS group, *n* = 8). ++, +++ *p* < 0.01, 0.001 vs baseline, respectively. CFA alone induced mechanical allodynia for 14 days after CFA injection (E2 + CFA + non-FS group, n = 8). #, ### *p* < 0.05, 0.001 vs baseline, respectively. The mechanical withdrawal threshold significantly decreased on day 3 to day 15 post FS stress in the E2 + CFA + FS rats compared with the sham treated rats. §, §§ *p* < 0.05, 0.01 vs the E2 + saline + non-FS group at the same time point, respectively. No mechanical allodynia was observed in the sham treatment group (E2 + saline + non-FS group, *n* = 8)

In oil treated group, there were significant differences among groups in the mechanical withdrawal threshold (two-way ANOVA, *F*_18,186_ = 2.985, *p* < 0.001 for interaction; *F*_6,186_ = 7.616, *p* < 0.001 for time factor; *F*_3,31_ = 1.049, *p* = 0.3850 for group factor, Fig. 3a). Sidak post-hoc tests showed that FS stress combined with orofacial inflammation induced mechanical allodynia persisted over 15 days post FS (*p* < 0.001 for day 3, *p* = 0.0082 for day 7, *p* < 0.001 for day 11, *p* = 0.0037 for day 15 respectively compared to baseline, *n* = 10). There were no significant nociceptive changes (*p* > 0.05 for all time points) in rats with FS alone (oil + saline + FS group, *n* = 9), CFA alone (oil + CFA + non-FS group, *n* = 8) or all sham treatment (oil + saline + non-FS group, *n* = 8). In E2 treated group, there were also significant differences among groups in the mechanical withdrawal threshold (two-way ANOVA, *F*_18,192_ = 4.330, *p* < 0.001 for interaction; *F*_6,192_ = 22.44, *p* < 0.001 for time factor; *F*_3,32_ = 3.998, *p* = 0.0159 for group factor, Fig. 3b). Sidak post-hoc tests showed that FS alone produced early changes on day 3 and day 7 post FS (E2 + saline + FS group, *p* = 0.0023 for day 3 and *p <* 0.001 for day 7 respectively compared to baseline, *n* = 8). CFA alone group induced mechanical allodynia for 14 days after CFA injection (E2 + CFA + non-FS group, *p* < 0.001 for day 6, day 10 and *p* = 0.0436 for day 14 after CFA injection respectively compared to baseline, n = 8). However, FS stress combined with orofacial inflammation resulted in prolonged allodynia over 15 days post FS (E2 + CFA + FS group, *p* < 0.001 for days 3 to 15 respectively compared to baseline, *n* = 11). No mechanical allodynia (*p* > 0.05 for all time points) was observed in sham treatment (E2 + saline + non-FS group, *n* = 8). When compared to the sham treated rats, the mechanical withdrawal threshold in FS stress combined with orofacial inflammation group significantly decreased (Fig. 3b). These findings suggest less effects of estrogen on stress-induced long-lasting mechanical allodynia in rats with orofacial inflammation.

**Fig. 4:**
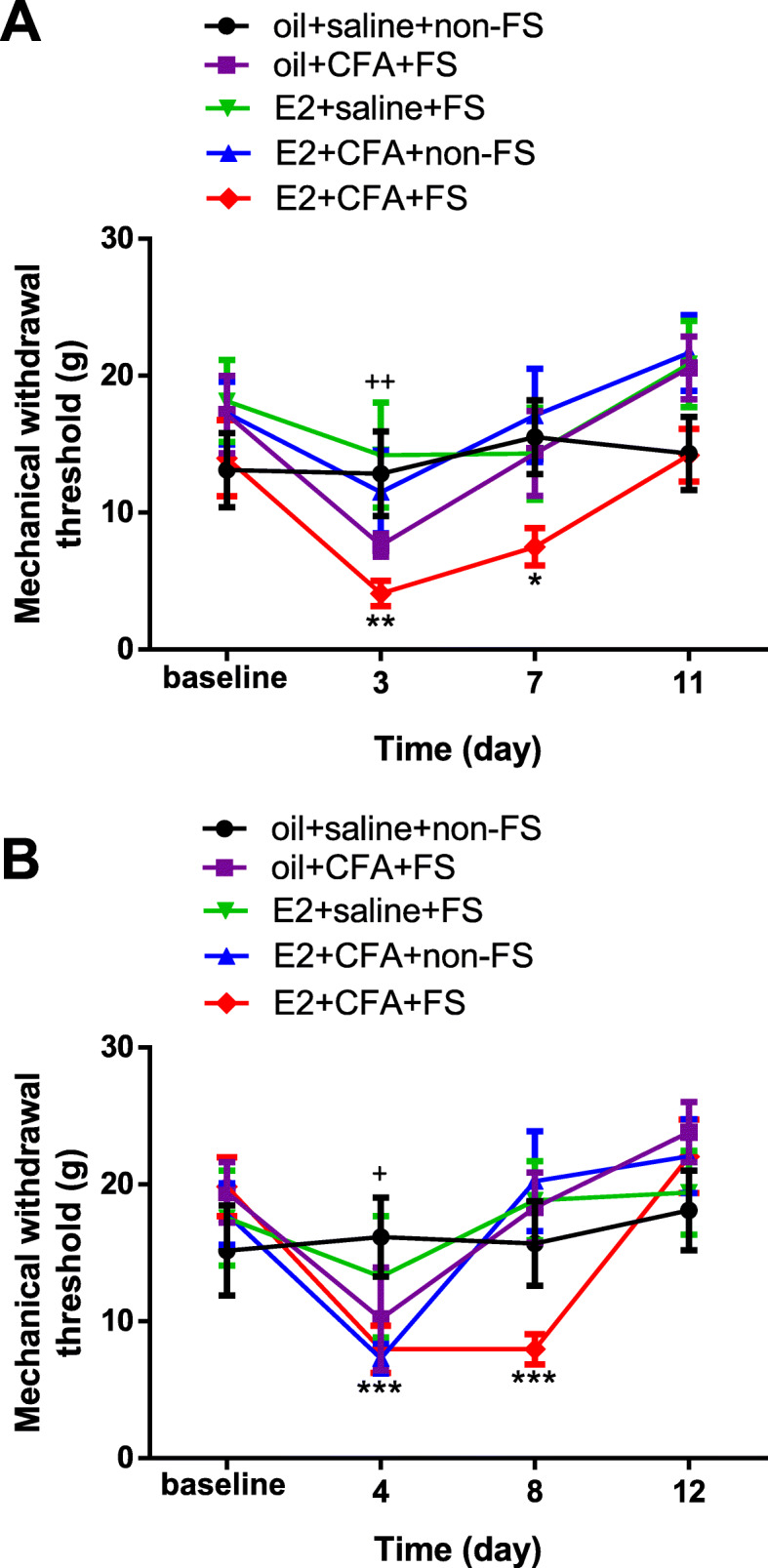
The mechanical allodynia in the upper back and thigh root occurred following 3 day FS in E2 treated rats with orofacial inflammation. **a** In the upper back, the mechanical allodynia persisted 7 days post FS in the E2 + CFA + FS rats (*n* = 8). *, ** *p* < 0.05, 0.01 vs baseline, respectively. In the oil + CFA + FS group, mechanical allodynia persisted till day 3 after FS (*n* = 7). ++ *p* < 0.01 vs baseline. No changes in the mechanical withdrawal threshold were found in the E2 + saline + FS group (*n* = 6), E2 + CFA + non-FS group (*n* = 6) and sham group (oil + saline + non-FS group, *n* = 7). **b** In the thigh root, the mechanical allodynia persisted 8 days after FS in the E2 + CFA + FS group (*n* = 8). *** *p* < 0.001 vs baseline. In the oil + CFA + FS group (*n* = 7), mechanical allodynia persisted till day 4 after FS. + *p* < 0.05 vs baseline. No changes in the mechanical withdrawal threshold were found in the E2 + saline + FS group (*n* = 6), E2 + CFA + non-FS group (*n* = 6) and sham group (oil + saline + non-FS group, *n* = 7)

In order to further demonstrate the wide-spreading pain happened in this comorbidity animal model, we also tested the mechanical thresholds in the upper back and thigh root in rats. There were significant differences among groups in the mechanical withdrawal threshold in the upper back (two-way ANOVA, *F*_12,84_ = 1.682, *p* = 0.0853 for interaction; *F*_3,84_ = 15.60, *p* < 0.001 for time factor; *F*_4,28_ = 1.752, *p* = 0.1667 for group factor, Fig. 4a). Sidak post-hoc tests showed that mechanical allodynia happened in the upper back persisted 7 days after FS in the E2 + CFA + FS group (*p* = 0.0012 for day 3 and *p* = 0.0486 for day 7 compared to baseline, *n* = 8). In contrast, mechanical allodynia only at earlier time point was observed in the upper back in the oil + CFA + FS group (*p* = 0.0019 for day 3 compared to baseline, *n* = 7, Fig. 4a). No changes in the mechanical withdrawal threshold (*p* > 0.05 for all time points) were found in the upper back in the E2 + saline + FS group (*n* = 6), E2 + CFA + non-FS group (n = 6) and sham group (oil + saline + non-FS group, *n* = 7).

Similarly, significant differences among groups in the mechanical withdrawal threshold in the thigh root were also found (two-way ANOVA, *F*_12,84_ = 2.705, *p* = 0.0039 for interaction; *F*_3,84_ = 16.51, *p* < 0.001 for time factor; *F*_4,28_ = 0.4034, *p* = 0.8046 for group factor, Fig. 4b). Sidak post-hoc tests showed that mechanical allodynia persisted 8 days in the thigh root in the E2 + CFA + FS group (*p* < 0.001 for day 4 and day 8 compared to baseline, *n* = 8). However, mechanical allodynia only on day 4 post FS was observed in the thigh root (*p* = 0.0117, *n* = 7, Fig. 4b) in the oil + CFA + FS group. No changes in the mechanical withdrawal threshold compared to baseline (*p* > 0.05 for all time points) were found in the thigh root in the E2 + saline + FS group (*n* = 6), E2 + CFA + non-FS group (*n* = 6) and sham group (oil + saline + non-FS group, n = 7). Taken together, these findings confirm that prolonged and wide-spreading hyperalgesia develop in female rats with orofacial pain after stress.

### GluN1 receptors are involved in stress-induced wide-spreading hyperalgesia in female rats with orofacial pain

**Fig. 5:**
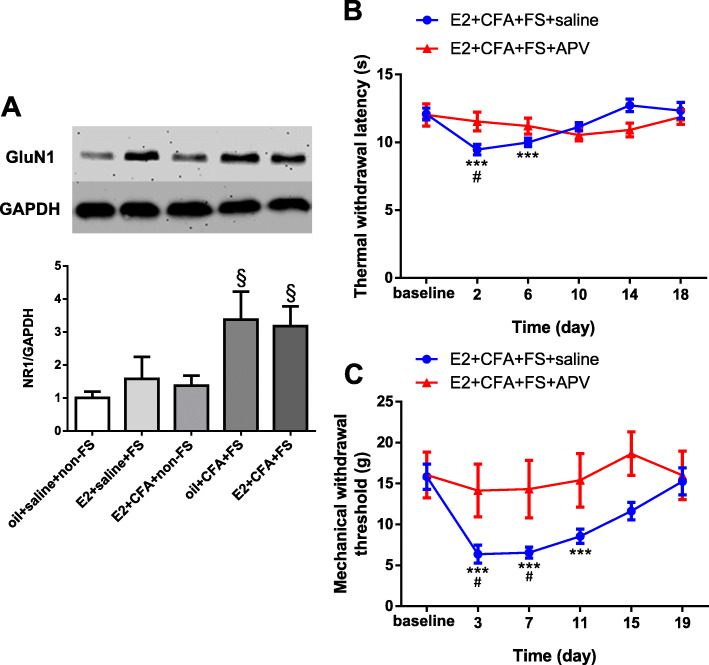
GluN1 receptors were involved in stress-induced wide-spreading hyperalgesia in female rats with orofacial pain. **a** The expression of GluN1 receptors in L4-L5 spinal dorsal horn in the E2 + CFA + FS group (*n* = 5) and oil + CFA + FS group (*n* = 5) significantly increased than that in the oil + saline + non-FS group (*n* = 5). § *p* < 0.05 vs the oil + saline + non-FS group. **b** NMDA receptor antagonist APV but not vehicle (saline) blocked the development of thermal hyperalgesia induced by stress in female rats with orofacial inflammation. *** *p* < 0.001 vs baseline. The thermal withdrawal latency significantly decreased on day 2 post FS stress in saline treated rats (E2 + CFA + FS + saline group, *n* = 12) compared with APV treated (E2 + CFA + FS + APV group, *n* = 8). # *p* < 0.05 vs the E2 + CFA + FS + APV group at the same time point. **c** APV but not vehicle blocked mechanical allodynia induced by stress in female rats with orofacial inflammation. *** *p* < 0.001 vs baseline. The mechanical withdrawal threshold significantly decreased on day 3 and day 7 post FS stress in saline treated rats (E2 + CFA + FS + saline group, *n* = 12) compared with APV treated (E2 + CFA + FS + APV group, n = 8). # *p* < 0.05 vs the E2 + CFA + FS + APV group at the same time point

To determine whether spinal NMDA receptors mediate stress-induced wide-spreading hyperalgesia in the hindpaw, we examined the protein expression of GluN1 receptors in the L4-L5 spinal dorsal cord, where is the projection site of primary afferent neurons innervating the hindpaws. The expression of GluN1 receptors significantly increased in the E2 + CFA + FS group (*p* = 0.017) and oil + CFA + FS (*p* = 0.0223) group compared to the oil + saline + non-FS group (*n* = 5 for each group, Fig. 5a). Next, we tested whether NMDA receptors were involved in the wide-spreading hyperalgesia induced by FS stress in E2 treated rats with masseter muscle inflammation. We found that intrathecal injection of NMDA receptor antagonist APV blocked both thermal hyperalgesia (two-way ANOVA, *F*_5,90_ = 5.569, *p* < 0.001 for interaction; *F*_5,90_ = 6.506, *p* < 0.001 for time factor; *F*_1,18_ = 0.01154, *p* = 0.9156 for group factor, Fig. 5b) and mechanical allodynia (two-way ANOVA, *F*_5,90_ = 3.637, *p* = 0.0048 for interaction; *F*_5,90_ = 7.985, *p* < 0.001 for time factor; *F*_1,18_ = 4.710, *p* = 0.0436 for group factor, Fig. 5c) in the E2 + CFA + FS + APV group compared to saline treated group (E2 + CFA + FS + saline group). These results indicate that stress-induced upregulation of GluN1 receptors contributes to wide-spreading hyperalgesia in female rats with orofacial inflammation.

### 5-HT_3_ receptors are involved in stress-induced wide-spreading hyperalgesia in female rats with orofacial pain

**Fig. 6:**
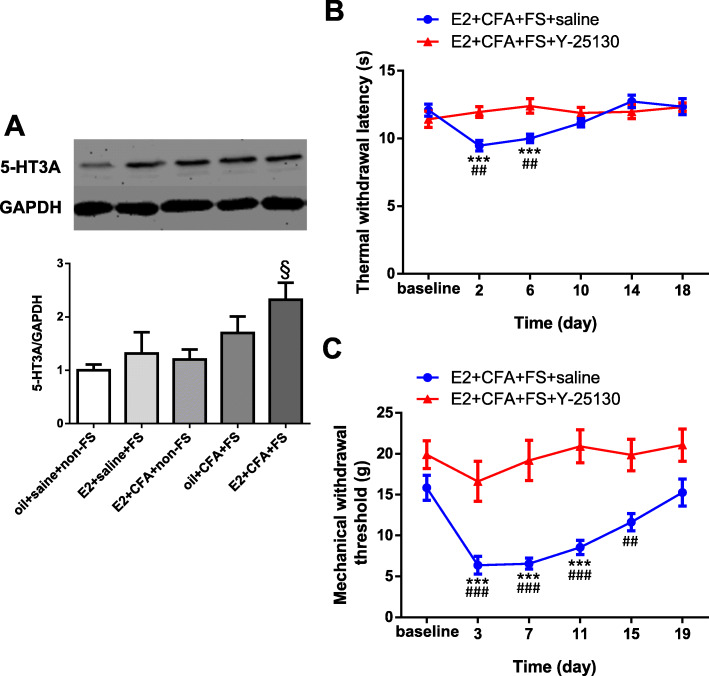
5-HT_3_ receptors were involved in stress-induced wide-spreading hyperalgesia in female rats with orofacial pain. **a** The expression of 5-HT_3A_ receptors in L4-L5 spinal dorsal horn in the E2 + CFA + FS group (*n* = 5) significantly increased when compared with that in the oil + saline + non-FS group (*n* = 5). § *p* < 0.05 vs the oil + saline + non-FS group. **b** 5-HT_3_ receptor antagonist Y-25130 but not vehicle blocked thermal hyperalgesia induced by stress combined with orofacial inflammation in female rats. *** *p* < 0.001 vs baseline. The thermal withdrawal latency significantly decreased on day 2 and day 6 post FS stress in saline treated rats (E2 + CFA + FS + saline group, *n* = 12) compared with Y-25130 treated (E2 + CFA + FS + Y-25130 group, *n* = 7). ## *p* < 0.01 vs the E2 + CFA + FS + Y-25130 group at the same time point. **c** Y-25130 blocked mechanical allodynia induced by stress in female rats with orofacial inflammation. *** *p* < 0.001 vs baseline. The mechanical withdrawal threshold significantly decreased for 15 days post FS stress in saline treated rats (E2 + CFA + FS + saline group, *n* = 12) compared with Y-25130 treated (E2 + CFA + FS + Y-25130 group, *n* = 7). ##, ### *p* < 0.01, 0.001 vs the E2 + CFA + FS + Y-25130 group at the same time point

Finally, to determine whether spinal 5-HT_3_ receptors, main target of 5-HT-dependent descending facilitation, contribute to stress-induced spreading pain, we examined 5-HT_3A_ receptor expression in the L4-L5 spinal dorsal cord because all functional 5-HT_3_ receptors require at least one 5-HT_3A_ subunit in the central nervous system (CNS). The Western blot data showed that the expression of 5-HT_3A_ receptors significantly increased in the E2 + CFA + FS group compared to the oil+saline+non-FS group (*p* = 0.0213, *n* = 5 for each group, Fig. 6a).

To determine whether enhanced 5-HT_3_ receptors contribute to stress-induced wide-spreading hyperalgesia in female rats with orofacial inflammation, intrathecal injection of 5-HT_3_ receptor antagonist Y-25130 was performed before CFA injection, each FS stress and 1 day post FS. Intrathecal injection of Y-25130 blocked the reductions in thermal withdrawal latency (two-way ANOVA, *F*_5,90_ = 6.065, *p* < 0.001 for interaction; *F*_5,90_ = 4.729, *p* < 0.001 for time factor; *F*_1,18_ = 3.655, *p* = 0.0720 for group factor, Fig. 6b) and mechanical withdrawal threshold (two-way ANOVA, *F*_5,85_ = 3.100, *p* = 0.0128 for interaction; *F*_5,85_ = 7.178, *p* < 0.001 for time factor; *F*_1,17_ = 45.88, *p* < 0.001 for group factor, Fig. 6c) following repeated FS stress in female rats with orofacial inflammation (E2 + CFA + FS + Y-25130 group) compared to saline treated group (E2 + CFA + FS + saline group). These results indicate that upregulation of spinal 5-HT_3_ receptors plays a role in stress-induced wide-spreading hypersensitivity in rats with masseter muscle inflammation.

## Discussion

In the present study, we demonstrated that repeated forced swim produces a long-lasting and wide-spreading somatic hyperalgesia in female rats with existing masseter muscle inflammation, which suggests that orofacial pain and estrogen are essentially synergetic factors for stress-induced persistent comorbid pain. Spinal GluN1 and 5-HT_3_ receptors are involved in central sensitization underlying the development of wide-spreading comorbid pain conditions.

CFA-induced masseter muscle inflammation has been used to mimic myofascial TMD (mTMD) [15]. Masseter muscle dysfunction is the initial stage of TMD, which accounts for a large proportion of TMD. The main clinical manifestations of TMD are persistent facial pain in patients, abnormal sensitivity to tactile stimuli, pain in the joint area, joint clicking and noise during exercise and jaw movement [16, 17]. Our previous study showed that injection of CFA into the masseter muscles increased mechanosensitivity in the orofacial region for 20 days post FS stress in E2 replacement rats [2]. Stress with repeated FS produced prolonged visceral hypersensitivity in rats with orofacial inflammatory pain but not normal animals, which may model the comorbidity of TMD and IBS in clinic [2]. The pathogenic factors and clinical manifestations of TMD, IBS and FMS are diverse [17]. However, a large number of studies have shown that these functional pain syndromes are closely related to mental factors such as stress [18–20]. TMD is characterized by persistent pain in the maxillofacial region and the most of FMS patients (75%) conform to the diagnostic criteria of TMD [21]. The symptoms of FMS in patients with mTMD are more severe and more prone to mental stress [22, 23]. Both of TMD and FMS have higher incidence in female [20, 24]. Because of this point, we used female rats to establish pre-clinical model to mimic the comorbidity of TMD and FMS.

The mechanisms for the comorbidity of TMD and FMS are unclear yet. Clinical treatment for this comorbidity is not optimistic and the unsatisfactory curative effect makes patients more anxious for their pain conditions. Therefore, it is particularly important to investigate the mechanism of this comorbid pain conditions and develop targeted drugs to efficiently prevent these functional pain syndromes. In the previous study, we found that stress could induce visceral hypersensitivity accompanied with mechanical allodynia in the lower back of female rats with orofacial inflammation [2]. In the present study, we used the same model and further observed behavioral hypersensitivity in the hindpaws, which is consistent with the time course of referred pain in lower back [2]. Although earlier mechanical hypersensitivity presented was observed only in the forepaw, this stress-induced pain spreading to the multiple parts of the body including the upper back and the thigh root indicates the development of wide-spreading somatic pain after stress in female rats, especially with orofacial pain. In fact, the main clinical manifestations of FMS are extensive diffuse musculoskeletal pain [25]. Therefore, mechanical pain tested in multiple areas of the body is helpful to completely reflect the pain sensitization of FMS.

In the present study, we focused on the impacts of estrogen and existing orofacial pain on the development of stress-induced somatic comorbid hyperalgesia. In our comorbid pain model, we developed 8 animal groups to examine the synergetic roles of E2, masseter muscle inflammation and FS in this comorbid pain model. Our data showed that all of three factors (E2, masseter muscle inflammation and FS) were essential and necessary for the development of long-lasting wide-spreading hypersensitivity including thermal and mechanical hyperalgesia. In addition, combination of FS and orofacial inflammation was sufficient to produce mild mechanical hyperalgesia. Injection of E2, CFA or application of FS alone could not induce somatic hyperalgesia in rats. Compared with the behavioral nociception in control rats, stress resulted in severe and prolonged somatic hyperalgesia only in female rats with masseter muscle inflammation. Replacement of E2 in OVx rats or existing pain in orofacial region did not affect basic nociception in somatic body. Therefore, each of these factors is indispensable to induce persistent somatic hyperalgesia. Importantly, although no effects on the duration of stress-induced mechanical allodynia, E2 treatment obviously enhanced wide-spreading nociceptive responses in rats with orofacial inflammation after stress. It is reported that repeated FS [26] or intermittent cold stress (ICS) [27] induces thermal hyperalgesia in the hindpaw in male mice. In the present study, however, we did not find that the same stress protocol changed mechanical and thermal nociception in OVx rats, suggesting that stress-induced pain also depends on physiological E2 level in normal rats. The evidence also supports our hypothesis that stress may consider as one of possible critical factors underlying the development of somatic pain in female rats. Therefore, these data strongly indicate that the concurrent incidence of both female hormone E2 and existing orofacial pain remains in the development of stress-induced wide-spreading pain conditions.

There is no pathological cause of FMS [28]. Recent study with functional neuroimaging has consistently demonstrated altered central pain perception in the brain of patients with FMS [5]. Accumulated studies have demonstrated that either orofacial inflammation or stress may affect the same areas specific for central pain processing and modulation [29, 30]. In our comorbid pain model, we hypothesize that stress signal input could concurrently enhance or prolong the masseter muscle inflammation-induced neuronal hyperactivity in supraspinal pain pathway, integrally resulting in central sensitization in the spinal dorsal horn through the descending brainstem modulatory system.

The classic descending pain modulatory system is mediated by the periaqueductal gray (PAG)-rostral ventrolateral medulla (RVM) neural circuitry, which regulates nociceptive transmission and processing in the spinal dorsal horn through the descending spinal projection from the RVM [31–34]. Active 5-HT-dependent descending pathway from the RVM plays a key role for the maintenance of persistent pain after tissue and nerve injury [6, 35, 36]. Spinal 5-HT_3_ receptors widely existing in the dorsal horn of spinal cord have been reported to mediate descending serotonergic facilitation and the upregulation of 5-HT_3_ receptor expression in the dorsal horn presents enhanced descending pain facilitation after tissue and nerve injury [6, 37, 38]. Some studies have realized the important role of peripheral 5-HT_3_ receptors in functional pain syndromes such as TMD and FMS [39, 40]. However, few studies focused on the role of spinal 5-HT_3_ receptors in the development of FMS. In recent years, our lab and others have shown that upregulation of 5-HT_3_ receptor expression in the spinal dorsal horn mediates 5-HT-dependent descending facilitation, which underlies the maintenance of neuropathic pain conditions including secondary hyperalgesia after tissue and nerve injury [6, 8, 41]. Based on these facts, the secondary purpose of the present study was to investigate whether 5-HT_3_ receptors in the spinal cord contribute to stress-induced wide-spreading pain in our comorbid pain model. Our data demonstrated the increased expression of 5-HT_3_ receptors in the lumbar dorsal horn after FS in female rats only with orofacial pain. Functional blockade of spinal 5-HT_3_ receptors totally prevented stress-induced thermal hyperalgesia and mechanical allodynia in the hindpaws. There are more than ten 5-HT receptor subtypes existing in the spinal cord and modulating nociceptive inputs and transmission. The effect of 5-HT on pain modulation in the spinal cord can be either inhibitory or facilitatory, depending on the receptor subtypes activated and functional diversity of 5-HT receptors [35, 42, 43]. Thus, we examined spinal 5-HT_3_ receptor expression in this model to unmask the central mechanisms mediating the development of somatic comorbid hyperalgesia through activating 5-HT-dependent descending facilitation.

NMDA receptor is a type of glutamate receptors mediating the functions of the excitatory transmitter glutamate in the CNS. Activation of NMDA receptors initiates intracellular cascades through calcium influx and activation of protein kinases, which in turn modulates cell membrane excitability and enhances nociceptive transmission [44]. The expression of NMDA receptors has been shown to be upregulated within the dorsal horn of spinal cord after peripheral injury, which contributes to the mechanisms of neuropathic pain [45, 46]. It has been reported that NMDA receptors are involved in the development of hyperalgesia under the mental factors such as anxiety, depression and stress [47, 48] and in the development of fibromyalgia [49]. Blocking NMDA receptors reduces hyperalgesia in TMD animal model [50]. Therefore, the regulation of NMDA receptors is the target of treatment intervention. The literature has demonstrated that NMDA receptors play a critical role in neuronal and synaptic hyperexcitability and long-term plasticity in the CNS. The GluN1 receptor subunit is a main component of functional and structural NMDA receptor complexes and is widely used as biomarker for NMDA receptors for examination of their neuronal expression and regulation. We previously showed that increased expression of GluN1 receptors in the spinal dorsal horn mediated 5-HT-dependent descending pain facilitation and the upregulation of 5-HT_3_ receptors [6]. As a biomarker of central sensitization in the spinal cord, we preferred to test the expression of GluN1 receptors in the spinal cord in our comorbid pain model without somatic tissue injury. The present data showed that the stress-induced somatic hyperalgesia in rats with orofacial pain was blocked by intrathecal administration of APV, indicating that spinal NMDA receptors may contribute to the central sensitization in this animal model.

Altogether, our current study suggests that active 5-HT-dependent descending facilitation and subsequent central sensitization in the spinal dorsal horn after stress may mediate central mechanisms underlying the development of wide-spreading somatic pain in our comorbid pain model.

## Conclusion

In conclusion, repeated FS stress induced somatic hyperalgesia in female rats with masseter muscle inflammation, which may explain the underlying CNS mechanisms of comorbid pain condition occurred in TMD and FMS patients. NMDA and 5-HT_3_ receptors play important roles in the occurrence and development of this somatic hyperalgesia. These results provide a theoretical basis for the development of drugs that modulate central 5-HT system to treat TMD and FMS comorbidity.

## Data Availability

The datasets generated and analyzed during the current study are available from the corresponding author on reasonable request.

## Abbreviations

5-HT: 5-hydroxytryptamine
APV: 2-amino-5-phosphonovaleric acid
BCA: bicinchoninic acid
CFA: Complete Freund’s adjuvant
CNS: Central nervous system
E2: 17β-estradiol
fMRI: Functional magnetic resonance imaging
FMS: Fibromyalgia syndrome
FS: Forced swim
IBS: Irritable bowel syndrome
ICS: Intermittent cold stress
mTMD: Myofascial TMD
NMDA: N-methyl-D-aspartic acid
OVx: Ovariectomized
PAG: Periaqueductal gray
RVM: Rostral ventrolateral medulla
TMD: Temporomandibular disorder
